# School food policy at Dutch primary schools: room for improvement? Cross-sectional findings from the INPACT study

**DOI:** 10.1186/1471-2458-13-339

**Published:** 2013-04-12

**Authors:** Wilke JC van Ansem, Carola TM Schrijvers, Gerda Rodenburg, Albertine J Schuit, Dike van de Mheen

**Affiliations:** 1 IVO Addiction Research Institute, Heemraadssingel 194, Rotterdam, DM, 3021, The Netherlands; 2 Erasmus Medical Centre, P.O. Box 2040, Rotterdam, CA, 3000, The Netherlands; 3 National Institute for Public Health and the Environment, Public Health and Health Services Division, PO Box 1, Bilthoven, BA, 3720, The Netherlands; 4 VU University Amsterdam, Department of Health Sciences and EMGO Institute for Health and Care Research, De Boelelaan 1105, Amsterdam, HV, 1081, The Netherlands

**Keywords:** Obesity, Children, School food policy, Food rules, Primary school, School health policy

## Abstract

**Background:**

Schools can play an important role in the prevention of obesity, e.g. by providing an environment that stimulates healthy eating habits and by developing a food policy to provide such an environment. The effectiveness of a school food policy is affected by the content of the policy, its implementation and its support by parents, teachers and principals. The aim of this study is to detect opportunities to improve the school food policy and/or implementation at Dutch primary schools. Therefore, this study explores the school food policy and investigates schools’ (teachers and principals) and parents’ opinion on the school food policy.

**Methods:**

Data on the schools’ perspective of the food policy was collected from principals and teachers by means of semi-structured interviews. In total 74 principals and 72 teachers from 83 Dutch primary schools were interviewed. Data on parental perceptions about the school food policy were based on a cross-sectional survey among 1,429 parents from the same schools.

**Results:**

Most principals (87.1%) reported that their school had a written food policy; however in most cases the rules were not clearly defined. Most of the principals (87.8%) believed that their school paid sufficient attention to nutrition and health. Teachers and principals felt that parents were primarily responsible to encourage healthy eating habits among children, while 49.8% of the parents believed that it is also a responsibility of the school to foster healthy eating habits among children. Most parents reported that they appreciated the school food policy and comply with the food rules. Parents’ opinion on the enforcement of the school food policy varied: 28.1% believed that the school should enforce the policy more strongly, 32.1% was satisfied, and 39.8% had no opinion on this topic.

**Conclusion:**

Dutch primary schools could play a more important role in fostering healthy eating habits among children. The school food policy could be improved by clearly formulating food rules, simplifying supervision of the food rules, and defining how to enforce the food rules. In addition, the school food policy will only influence children’s dietary behaviour if both the school and the parents support the policy.

## Background

Childhood obesity is a major public health concern in many countries [1,2]. Globally, in 2010 more than 40 million children aged ≤ 5 years were overweight [3]. In the Netherlands, obesity is also a health concern; not necessarily because the prevalence rates are high (they are lower compared with some other European countries [4,5]), but mainly because the prevalence rates of overweight among Dutch children and adolescents have increased over the years. Among Dutch boys (aged 2–21 years) the prevalence rate of overweight almost tripled from 5.1% in 1980 to 13.3% in 2010, and for Dutch girls these rates increased from 7.2% in 1980 to 14.9% in 2010 [6]. Overweight and obesity are related to a range of both short and long-term negative health outcomes such as hypertension, diabetes mellitus type II, pulmonary disorders and cardiovascular disease [7-10]. Furthermore, childhood obesity tracks into adulthood [11]. One of the main causes of obesity is unhealthy dietary behaviour, such as high consumption of sugar-sweetened beverages, large portions of food, and skipping meals [12]. Eating habits established in childhood also track into later life [13,14]. Therefore, it is important to study the determinants of eating habits of children and develop interventions and policies to establish healthy dietary habits at an early stage.

Parents have a strong influence on children’s dietary behaviour and the development of their eating habits [15-17]. For example, parents determine the availability and accessibility of food at home, set rules and regulations concerning food consumption, and also act as role models.

The school is also considered an important place for the prevention of childhood obesity as children spend a significant part of their time there and often consume food/beverages during school time [18]. Moreover, school staff are able to reach both children and parents, and schools can be an environment that stimulates healthy eating habits [19]. A school food policy can be the basis of such a healthy eating environment. School food policy is a broad concept and includes nutrition guidelines (e.g. nutrition standards for menu planning), regulation of the availability of food and beverages at schools (e.g. types of food sold in the school canteen or vending machines), food rules (e.g. rules about the types of food children are allowed to consume during school time), and price interventions regarding food and beverages sold in school canteens (subsidised provision of specific foods or control of the price of foods and beverages for sale to children). Several studies examined the effectiveness of a school food policy. For example, Jaime et al. reviewed the effectiveness of school food policies in the USA and Europe (focusing on nutritional guidelines, regulation of food and beverages availability and price interventions) with regard to improving the school food environment, children’s dietary intake, and decreasing overweight/obesity of children. In that review, most evidence for effectiveness was found for the impact of nutritional guidelines, i.e. 8 out of 9 studies showed a positive change in decreasing fat intake and increasing fruit and vegetable availability at school [20]. A Belgium-Flanders study examined the influence of the school food policy (availability of food items, food rules, nutrition education program) on the consumption of fruit, soft drinks, crisps and sweets at primary and secondary schools. That study showed that only at secondary schools did the school food policy have an impact on adolescents’ consumption of soft drinks, crisps and sweets [21]. Moreover, it was found that school-based interventions can improve the dietary behaviour of children. For example, Van Cauwenberghe et al. reviewed school-based interventions across Europe and found strong evidence that multi-component interventions that combine improved availability of fruit and vegetables with a nutrition education curriculum delivered by the teacher and at least some parental involvement can improve children’s fruit and vegetable consumption [22]. De Bourdeaudhuij et al. reviewed school-based interventions that combined nutrition and physical activity approaches among children and adolescent’s in Europe and concluded that multi-component interventions might be preferable in school-based nutrition and physical activity interventions to reduce obesity in European children and adolescents [23].

The Dutch school system is different from school systems in other countries, both in general and regarding food policy. In the Netherlands, compulsory education starts at age 5 years, but in practice most children start school at age 4 years. Children aged 4–12 years attend primary school (some primary schools differentiate between infant school and junior school). In the Netherlands, schools are autonomous regarding fostering a healthy food environment and there is no legal obligation to implement a school health promotion program or food policy. Because almost no primary schools offer school meals, Dutch primary schools play a smaller role in fostering healthy eating habits among children than in countries where schools offer cooked meals during the lunch breaks. On the other hand, many Dutch primary schools implement school food rules (e.g. ‘children are not allowed to drink sugar-sweetened beverages during school time’) and have some kind of food policy.

As stated before, schools are considered as important settings to promote healthy eating habits among children and a school food policy can be an important tool to create a healthy eating environment at school. Although a range of studies has focussed on the development and evaluation of school-based interventions [22-24] only a few studies have explored the food environment and school food policies at primary schools [20,21], while such knowledge is required to improve the school food environment. The effectiveness of school food policies depends on the content of the policy and on the way it is implemented. Furthermore, the support of teachers, principals and parents is essential for the policy to work, as all are stakeholders in childhood obesity prevention. Insight in the perspectives of the schools (teachers and principals) and of the parents regarding the school food policy can provide valuable knowledge that can be used to improve the policy.

The overall aim of this study is to explore opportunities to improve the school food policy and/or implementation of the policy in the Netherlands. Therefore, we describe the presence, content and implementation of the school food policy (e.g. communication, enforcement and compliance with the policy). Furthermore, this study explores schools’ (teachers and principals) and parents’ opinion on the school food policy (including appreciation of the policy and the role that schools play in fostering healthy eating habits among children).

### School food environment of Dutch primary schools

Before describing the study methods, we briefly outline the school food environment of Dutch primary schools. In the Netherlands, children bring their own food to school. Children have two breaks during a school day, i.e. a short break in the mid-morning and a lunch break. For both breaks, children have to bring their own packed food and beverages. During the morning break, it is normal for children to consume a small snack and/or beverage. During the lunch break, children either go home for lunch or they bring a packed lunch to school and remain at school. In the Netherlands it is not usual to have a cooked meal during the lunch break, most people have sandwiches and dairy drinks, coffee or tea.

At Dutch primary schools, no vending machines or canteens are available. Furthermore, the children are not allowed to leave the school premises during school time (as stated before children are allowed to go home for lunch; however children who remain at school during lunch break are not allowed to go to shops or cafes during that break). Classroom celebrations of a child’s birthday are common at Dutch primary schools: on such an occasion a child treats his/her classmates with e.g. fruit, cake or sweets and/or with a small gift.

## Methods

We studied a broad range of aspects related to school food policy, such as the content of the policy, communication about the policy with children and parents, compliance with the policy by parents and children, enforcement of the policy by the school, and appreciation of the policy by parents. In addition, we investigated the attention schools paid to the subject ‘nutrition and health’ in their curriculum, and the opinion of teachers, principals and parents regarding the role that schools play in the encouragement of healthy eating habits among children. In the present study, school food policy mainly refers to food rules (e.g. ‘children are not allowed to drink sugar-sweetened beverages during school time’), or recommendations (e.g. ‘we prefer that children eat fruit during the morning break’), which reflect the food environment at primary schools in the Netherlands.

### Study design

Data for this study were collected as part of the INPACT study (IVO Nutrition and Physical Activity Child cohorT), a longitudinal study on children’s nutritional behaviour and physical activity. INPACT started in 2008 among 8-year-old children and their parents. Data collection took place annually; the last wave of the study took place in 2011. Participants were recruited through primary schools in the southern parts of the Netherlands. In this area, all general primary schools (n = 265) were invited by the Municipal Health Services and 91 (34.3%) primary schools agreed to participate. In total 1844 (62.5%) primary caregivers gave informed consent. The INPACT study, including the procedure for informed consent, was approved by the Medical Ethical Committee of the Erasmus Medical Centre Rotterdam.

A mixed-method approach for data collection and analyses was used. Data on the schools’ perspective on school food policy were collected by means of semi-structured interviews with principals and teachers. Data on parental perspective of the school food policy were collected by means of a questionnaire.

### Interviews

Semi-structured face-to-face interviews were conducted with principals and teachers in the autumn of 2010 (third wave of the INPACT study). For each participating school in the INPACT study, we approached one principal and one teacher (fifth grade) to participate in the interviews. After they had provided informed consent, an appointment was made for the interview. We decided to interview fifth-grade teachers, because the participating children of the third wave of the INPACT study were in the fifth grade. Six trained interviewers visited the schools and conducted the interviews using a structured topic list (Table 1). The interview with the principal focused on the food rules, communication about food rules with children and parents, and attention paid to nutrition and health by the school. The interviews with the teachers focused on the topics concerning compliance with the school food rules by parents and children, enforcement of the school food rules, and appreciation of the school food rules by parents.

**Table 1:** Overview of the interview topics

**Topic**	**Principal**	**Teachers**
**School food policy**	- Does the school have rules/recommendations about the food and beverages children are allowed to consume during school time?	
	- Can you describe these food rules/recommendations?	
**Communication about food policy**	- Are the food rules/recommendations written down?	
	- Where are these food rules/recommendations written down?	
	- Do you inform parents about the food rules/recommendations?	
	- How do you inform parents about the food rules/recommendations?	
**Compliance with food policy**		- Does it frequently happen that children bring food to schools which is not allowed?
**Enforcement of food policy**		- What do you do when children bring food to school which is not allowed according to the food policy?
**Appreciation of food policy**		- Do you think that parents appreciate the school food policy?
		- Why do think that parents appreciate the school food policy?
**Attention paid to school food policy and nutrition education**	- Does the school pay attention to nutrition and health during class?	
	- Which method or program does the school use to educate children about nutrition and/or health?	
	- In your opinion, does the school pay enough attention to nutrition and health?	
**Schools’ role in encouragement healthy eating habits children**	- Do you believe that encouraging healthy food habits among children is a responsibility of the schools?	- Do you believe that encouraging healthy food habits among children is a responsibility of the schools?
	- What is the role of the parents in encouragement of healthy eating habits among children?	- What is the role of the parents in encouragement of healthy eating habits among children?

### Parental questionnaire

Data from parents were collected in the fourth wave of the INPACT study (autumn 2011); 1,429 (77.5%) primary caregivers completed a self-administered questionnaire at home. The questionnaire covered the following items: school food rules; compliance with school food rules, appreciation of the school food rules, enforcement of school food rules, and parents’ opinion about the role the school plays in the encouragement of healthy eating habits among children. Table 2 provides a detailed description of these items.

**Table 2:** Parents’ perspective on aspects of the school food policy

	**N**	**%**
**School food rules**		
*“Does your child’s school have rules about the food and drinks children are allowed to consume during…”*		
*Morning break*		
Yes	1108	78.2
No	216	15.3
Unknown	92	6.5
*Birthday celebration*		
Yes	631	44.9
No	620	44.1
Unknown	155	11.0
*Lunch break*		
Yes	606	42.8
No	431	30.5
Unknown	378	26.7
Compliance with school food rules *		
*“Do you comply with the food rules?”*		
*Morning break*		
Always	905	81.8
Not always	202	18.2
*Birthday celebration*		
Always	453	72.5
Not always	172	27.5
*Lunch break*		
Always	531	88.6
Not always	68	11.4
Reasons for non-compliance with school food rules**		
*“Why do you not always comply with the school food rules?”*		
*Morning break*		
Nothing else available at home	78	37.9
Almost no-one sticks to food rules	11	5.4
Child takes his/her own snack	36	17.5
Not always aware of rules	20	9.7
Disagree with rules	16	7.8
Other reason	45	21.8
*Birthday celebration*		
Almost no-one sticks to food rules	42	22.7
Not always aware of rules	14	7.6
Disagree with rules	19	10.3
Wishes child	70	37.8
Other reason	40	21.6
*Lunch break*		
Nothing else available at home	17	25.0
Almost no-one sticks to food rules	4	5.9
Child takes his/her own snack	6	8.8
Not always aware of rules	11	16.1
Disagree with rules	14	20.6
Other reason	16	23.5
Enforcement of school food rules***		
*“ I believe that my child’s school should more strongly enforce the food rules for morning breaks and classroom celebrations”*		
Agree	325	28.1
No opinion	460	39.8
Disagree	371	32.1
Appreciation of food rules***		
*“I appreciate that my child’s school has food rules about the food children are allowed to eat during morning breaks”*		
Agree	943	76.5
No opinion	230	18.7
Disagree	59	4.8
*“I appreciate that my child’s school has food rules about the food children are allowed to offer during classroom celebrations”*		
Agree	618	60.0
No opinion	336	32.6
Disagree	76	7.4
Role of the school in fostering healthy eating habits among children***		
**“***it is a responsibility of the school to foster healthy eating habits among children”*		
Agree	696	49.8
No opinion	454	32.5
Disagree	247	17.5

* Answering categories ranged from “yes, always” to “no, never” on a 5-point scale. For the purpose of analysis the variables were classified into “always” (‘yes, always) versus “not always” (‘yes, usually’, ‘no, usually not’, ‘sometimes’ and ‘no, never’).

** Multiple answers possible.

***Answering categories ranged from “completely agree” to “completely disagree” on a 5-point scale. For the purpose of analysis the variables were classified into “agree” (‘completely agree’ and ‘agree’); “no opinion” and “disagree” (‘disagree’ and ‘completely disagree’).

### Analysis

For each interview, a detailed summary was made using the interview transcripts and audiotapes. The detailed summary was organised according to the interview schedule. Data were analysed manually. Statements were independently coded by two researchers (GR and WVA). If there was any disparity between the coding of the researchers, the interpretation of the statement was discussed until consensus was reached. Categorical responses were counted. Descriptive statistics were used to examine parental perceptions about the school food policy.

## Results

A total of 72 teachers and 74 principals from 83 primary schools were interviewed. Most teachers were female (63.9%) and most principals were male (64.9%). The majority (92.1%) of the parental questionnaires was completed by the mother.

### School food policy

The majority of the principals reported to have a food policy, meaning that their school had food rules or recommendations about the food and/or beverages children were allowed to consume during school time. More than half of the principals (57.1%) reported that their school had food rules; 37.1% of the principals reported that their school had recommendations and 5.7% of the principals reported that their school had no food policy (Table 3). The most frequently mentioned specific rules or recommendations referred to the following subjects: 1) fruit and vegetables; 2) biscuits; 3) candy; 4) drinks, and 5) the amount of food and drinks children consumed. The rules or recommendations were in most cases unclearly defined. Especially the rules on biscuits were vaguely defined, as the most frequently mentioned definition of the rule on biscuits was: ‘*only healthy biscuits’/’no unhealthy biscuits’*. Furthermore, principals found it difficult to determine which foods are considered as healthy and which types of food are not. Some principals reported that their school had different rules or recommendations for younger children (age 4–6 years) and older children (age 7–12 years). For example, for younger children it was more often obligatory to eat fruit during morning breaks than for the older children. The above-mentioned rules or recommendations were only applicable to the morning break and lunch break. Concerning birthday celebrations, only five principals reported that their school had rules about the type of treats for these occasions. Most schools recommended ‘healthy’ treats, but this was not a rule.

**Table 3:** Schools’ perspective on aspects of the school food policy

	**Principals**		**Teachers**	
	**N**	**%**	**N**	**%**
**School food policy (n = 70)**				
Rules	40	57.1		
Recommendations	26	37.1		
No food policy	4	5.7		
**Written food policy (n = 64)**				
Yes	57	89.1		
no	7	10.9		
Activities undertaken to inform parents about food policy (n = 62)*				
School magazine	46	74.2		
Informal meetings	35	56.5		
Parent’s evening	5	8.1		
Parent’s council	2	3.2		
Interview on admission to school	7	11.3		
**Compliance with school food policy (n = 57)**				
Yes			45	78.9
Varying			5	8.7
No			7	12.2
Enforcement of school food policy (n = 62)*				
Addressing child			19	30.6
Addressing parent			8	12.9
Addressing both parent and child			27	43.5
Addressing in classroom			6	9.7
Child not allowed to consume the unhealthy food			8	12.9
No set approach			6	9.7
**Parental appreciation of school food policy (n = 62)**				
Good			54	87.1
Varying			7	11.2
Poor			1	1.6
**Attention for nutrition by the school (n = 74)**				
Insufficient	6	8.1		
Sufficient	65	87.8		
No opinion	3	4.1		
**Role of the school in fostering healthy eating habits among children (principals n = 73; teachers n = 72)**				
Parents primarily responsible, school supportive role	54	74.0	64	88.9
School and parents equally responsible	12	16.4	4	5.6
School no responsibility	7	9.5	4	5.6

* Multiple answers possible.

Parents were asked if the school had food rules for morning breaks, lunch breaks and classroom celebrations. The majority of the parents reported that the school of their child had rules about the food and drinks children were allowed to consume during the morning break (78.2%). Furthermore, 44.9% of the parents reported that the school had rules about birthday celebrations and 42.8% reported that the school had rules about the food and drinks children were allowed to consume during lunch break (Table 2).

### Communication about school food policy

Most schools (89.1%) had a written food policy. Schools also undertook activities to inform and remind parents about the school food policy. Most frequently mentioned were “reminder in school magazine” and “reminder in informal meetings”. The informal meetings with parents usually took place in the beginning of the school year. In some schools (n = 6; 17.1%) these informal meetings were only for parents of children at infant schools (aged 4–6 years) (Table 3).

### Compliance with school food policy

Most teachers (78.9%) reported a good compliance of parents with the school food policy, meaning that most parents and children abided by the food rules/recommendations (Table 3). However, it was mentioned that monitoring compliance with the school food policy was difficult because children often eat their snacks while playing outside in the school playground.

The majority of parents reported that they comply with the school food rules with respect to the morning break (81.8%), birthday celebration (72.5%) and lunch break (88.6%). The most frequently mentioned reason why parents did not comply with the food rules for the morning and lunch break food/beverages was *“nothing else available at home”.* In the case of birthday celebration, the most frequently mentioned reason for breaking the rule was “*preference of the child for another birthday celebration”* (Table 2).

### Enforcement of school food policy

It is not clear how often the school food policy was enforced. Difficulties in enforcement of the food policy mentioned by the teachers include unclearly defined food rules and difficulties in supervision. The most frequently mentioned approaches to enforce the food rules were “addressing/speaking to the child” and “addressing/speaking to both parents and child” in the case of lack of compliance with the rules by the child (Table 3).

Some parents were divided in their opinion about the enforcement of the school food rules regarding celebration treats and morning break snacks. The majority of parents (39.8%) had no opinion about the enforcement of the food rules, while others (28.1%) believed that the school food policy should be more strongly enforced, and 32.1% was satisfied with the enforcement of school food rules (Table 2).

### Appreciation of school food policy

Most teachers (87.1%) believed that the majority of the parents appreciate the school food policy (Table 3). The most frequently mentioned reasons why they thought that parents are satisfied with the food policy were “receive no complaints from parents”, “good compliance with the food rules” and “receive positive responses to the food rules”.

Most of the parents reported that they appreciated the school food rules regarding the morning breaks (76.5%) and birthday celebrations (60.0%). Nevertheless, 32.6% of the parents had no opinion about whether or not they appreciate that the school had food rules regarding birthday celebrations (Table 2).

### Attention paid to school food policy and nutrition education

Almost all schools educated children about nutrition. Only three principals reported that their school did not educate children on this topic. Education on nutrition was part of the school curriculum in the vast majority of the schools (reported by 85.1% of the principals). Nutrition education was embedded in educational topics like biology, physics or lifestyle. Also, 68.9% of the principals reported that their school participates in specific nutrition projects.

The majority of the principals (87.8%) believed that their school paid sufficient attention to nutrition and a healthy lifestyle. Nevertheless, several principals felt that the attention for nutrition and a healthy lifestyle could be improved (Table 3). Time was mentioned as a limiting factor to increase focus on this subject. Some principals argued that the socio-economic status of the school population influences the amount of attention the school paid to nutrition and a healthy lifestyle. According to these principals, schools in less deprived areas or schools with more highly educated parents paid less attention to nutrition and healthy lifestyle. The rationale given for this was that higher educated parents were more healthy-minded and had more knowledge about healthy eating behaviours and lifestyle and therefore the school could pay less attention to this subject.

### School’s role in encouraging healthy eating habits among children

The vast majority of teachers (88.9%) and principals (74.0%) felt that the parents were primarily responsible for encouraging healthy eating habits among children, with schools having a supportive role. In addition, 16.4% of the principals reported that in their opinion schools and parents are equally responsible to foster healthy eating habits among children (Table 3).

About half of the parents (49.8%) believed that encouraging healthy eating habits among children is also a responsibility of the school, 32.4% had no opinion about this topic, and 17.7% of the parents did not believe that the school should play a role in fostering healthy eating habits among children (Table 2).

## Discussion

This study investigated schools’ and parents’ perspective about the school food policy in Dutch primary schools. The study shows that most primary schools paid attention to nutrition and health. Most schools had a written food policy and informed parents about the food rules. Furthermore, in most schools education on nutrition was part of the school curriculum and most schools participated in specific nutrition projects. In general, parents appreciated the school food rules. Most principals believed that they had only a supportive role to foster healthy eating behaviour among children and considered parents to be primarily responsible. About half of the parents believed that schools should play a role in encouraging healthy food habits among children.

Only few studies have examined schools’ or parents’ view on the school food policy. For example, a USA study among teachers and parents of middle-school students found that both parents and teachers were concerned about the school food environment and believed that schools should offer students healthier food and beverages and limit low-nutrient food products [25]. However, the results of that study are not comparable with ours, since the school food environment at Dutch primary schools is very different (e.g. at Dutch primary schools no school meals are offered). A European study also found that parents and teachers considered the school food policy to be important, and the majority agrees that there should be a school policy restricting consumptions of snacks and soft drinks [26].

The majority of the principals reported that their school had a food policy: 57.1% reported to have food rules and 37.1% of the principals reported that their school had recommendations about the food and/or beverages children were allowed to consume during school time. Surprisingly, even more parents (78.2%) reported that the school of their child had rules about the food and beverages children were allowed to consume during the morning break. An explanation for the differences in reported school food rules by principals and parent could be that in the semi-structured interviews with principals and teachers a distinction was made between “rules” and “recommendations”, while the parent questionnaire did not. It is possible that parents reported that the school of their child had food rules, while the school in fact had recommendations. Another notable difference concerns the reported food rules for the morning break and for the lunch break by parents. Of the parents, 72.8% reported that their child’s school had rules about the food and drinks that children were allowed to consume during the morning break, but only 42.8% of the parents reported food rules for the lunch break. An explanation for this may be that some of the parents are not aware of the rules about the food and beverages that could be consumed during lunch break, because their child goes home for lunch.

However, it is a positive sign that the majority of the primary schools in our study sample had a written food policy and informed parents about the policy. However, having a food policy does not ensure monitoring and compliance with the policy. The content of the policy, implementation, communication, enforcement and support of stakeholders are important factors that influence the effectiveness of a policy. Our results show that the food rules were not always clearly formulated. Some schools argued that they had food rules while other schools referred to ‘recommendations’. In daily practice, however, there was little difference between so-called rules or recommendations about food and beverages. However, due to the vaguely formulated food rules, enforcement of these rules is difficult. Well-formulated rules provide clarity and help to ensure their enforcement. In addition, most schools had no clear approach of how to deal with children who brought food to school that was not allowed in school. In most cases the approach depended on the supervising teacher. Furthermore, supervision was difficult because children often eat their snacks while playing outside.

It is remarkable that most schools did not have food rules regarding birthday celebrations. Several USA studies reported that classroom celebration treats consist of low-nutrient calorie-dense snacks [27]. For example, the mean calorie intake during classroom celebrations for first-grade school children was estimated at 550 ± 212 calories [28]. Also, in the Netherlands, most birthday celebration treats are low-nutrient calorie-dense snacks such as potato chips, candy bars and cake [29]. Because on average there are ± 20–30 children in a classroom, a classroom celebration occurs regularly. Some principals stated that children at an infant school (children aged 4–7 years) sometimes offered fruit as a birthday celebration, however older children (junior school) rarely chose fruit for a birthday celebration. A possible explanation for this difference might be peer pressure: i.e. fruit or healthy snacks do not have a ‘cool’ image. However, birthday celebrations are a tradition at primary schools in the Netherlands and schools apparently have difficulty in adopting a restrictive policy regarding treats offered in birthday celebrations. A restrictive policy may be a promising way to provide a more healthy school food environment, because birthday celebration treats are mostly low in nutrient and high in calories, and such foods are also provided during other occasions at school, such as sports events, Christmas and other holiday celebrations.

Also notable is the difference between infant and junior schools: some principals mentioned that at infant school it was more obligatory for children to eat fruit during morning breaks. Also there was more supervision regarding the foods and beverages children bring to school (most younger children eat their morning break snack in the classroom). Some of the schools organised meetings to inform parents about school issues including the food policy, these meetings were only for parents of children at infant school and not for parents of children at junior school. An explanation for these differences may be that schools expect children and parents in junior schools to be more familiar with the food policy and thus better comply with the food rules during the remainder of their stay at school. However, schools could be more active and structured in bringing the food policy to the attention of parents and children, especially in junior school where peer pressure may increase.

The majority of principals and teachers believed that parents are primarily responsible for the acquisition of healthy eating habits in children and that schools play a supportive role in fostering children’s healthy eating habits. However, schools should consider themselves fully responsible to encourage healthy eating habits among children during school time. Dutch primary schools could be more involved in fostering healthy dietary behaviour among children by offering school meals, as often occurs in the UK and other countries; however, in the Dutch culture this may be difficult to implement. In addition to the school, parents can also take responsibility for fostering healthy eating habits among their children by, for example, supporting the school food policy.

Besides schools and parents, the government can also play a role in encouraging school food policies and a healthy eating environment at schools. Governments can encourage schools to have a food policy, to provide programs to stimulate healthy foods, set nutritional standards for the foods that are available at schools or provide national lunch programs. For example, national distribution schemes which provide free fruit and vegetables to children at schools have been implemented in the UK and USA [30,31]. Several years ago, the Australian government recommended that all primary schools implement a fruit and vegetable program (called ‘Crunch&Sip’) that provides a time in the class that children consume fruit and vegetables they bring from home [32]. Also in the Netherlands, there are some programs, such as *Schoolgruiten* (an initiative of the Dutch government in cooperation with other stakeholders such as The Netherlands Nutrition Centre Foundation), which is a program to stimulate children to eat fruit or vegetables during the morning break [33]. Governments can provide and support initiatives to improve the school food policy and environment; however the success of such initiatives depends on the adaptation and implementation of all relevant stakeholders including the school staff and parents.

### Strengths and limitations

A major strength of this study was the use of data from multiple sources. We collected data on the school’s perspective from principals and teachers, as well as data on the parental perspective. Furthermore, the sample was relatively large: we interviewed 74 principals and 72 teachers from 83 schools and 1,429 parents completed the parental questionnaire.

A limitation of this study is that not all data were collected in the same year. Data of parents were collected one year after the interviews with the principals and teachers took place. It is possible that the school food policy had changed during that year, but we believe that this is unlikely. Data from the teachers/principals and the data from the parents were collected from the same schools, but not compared at school level due to the low numbers of respondents per school. Another limitation is that this study does not provide insight into whether the school food policy actual improves the dietary behaviour of children at school. Although, this study identifies some opportunities to improve school food policy, future research should examine the influence of various aspects of school food policy on children’s actual dietary behaviour. Nevertheless, this study provides some valuable knowledge about schools’ and parents’ opinion on school food policy at Dutch primary schools. A final limitation is that the response rate of schools participating in the INPACT study was 34.1% (n = 91). The most frequently given reason for not participating in the INPACT study was a busy curriculum with a focus on the attainment targets (language skills and arithmetic/maths) of primary schools set by the government. The response rate of urban and rural schools was the same. Furthermore, the sample of schools in the INPACT study reflects the variety of schools in the Dutch primary school system and contains religious schools, public schools and schools based on various educational movements (e.g. Steiner Waldorf education or Montessori schools). A rather low school response rate may impact the generalizability of the results. However, we have no reason to believe that the non-response among schools had an important effect on our results because their refusal was not connected with the subject of the presented study (at the time of recruitment for the INPACT study this sub-study had not yet been designed). Furthermore, 91.3% of the participating schools in the INPACT study took part in the interviews which is a high response rate. Moreover, 77.5% of the parents participate in this study.

## Conclusion

This study investigated schools’ and parents’ perspective of the school food environment to detect opportunities to improve the school food policy. Most primary schools in this study had a school food policy and parents generally appreciated the school food rules. However, improvement is possible: schools should formulate clear rules about which food and beverages may be consumed during school time. Since teachers and principals may find it difficult to determine which food products are healthy and which are not, we recommend schools to cooperate with nutrition experts (e.g. dieticians, or The Netherlands Nutrition Centre Foundation). Within the food policy, special attention should be paid to birthday celebrations. In addition, supervision of the food and beverages that children bring to school can be simplified if children have to consume their food and/or beverages inside the classroom instead of while playing outside. Furthermore, schools should also clearly define how the school food policy will be enforced and the policy should be enforced by the entire school staff, rather than relying on teacher-dependent enforcement.

Finally, school food policy will only have an impact on children’s dietary behaviour if it is fully embraced by both the school and the parents. Dutch primary schools could play a more a significant role in fostering healthy dietary behaviour among children. However, teachers and principals, as well as parents, should take more responsibility in encouraging healthy eating habits among children and should fully support a school food policy.

## Competing interests

The authors declare that they have no competing interests.

## Authors’ contribution

WJCvA, CTMS and DvdM, were involved in the design of this study. WJCvA was responsible for data collection, performed the statistical analyses and drafted the manuscript. CTMS was the daily supervisor of the project. CTMS and DvdM helped with the interpretation of the data. GR participated in the data analyses and revised the manuscript. CTMS, JAS and DvdM helped to draft the manuscript. All authors read and approved the final manuscript.

## Pre-publication history

The pre-publication history for this paper can be accessed here:

http://www.biomedcentral.com/1471-2458/13/339/prepub
